# Biological activities of histidine-rich peptides; merging biotechnology and nanomedicine

**DOI:** 10.1186/1475-2859-10-101

**Published:** 2011-12-02

**Authors:** Neus Ferrer-Miralles, José Luis Corchero, Pradeep Kumar, Juan A Cedano, Kailash C Gupta, Antonio Villaverde, Esther Vazquez

**Affiliations:** 1 Institut de Biotecnologia i de Biomedicina, Universitat Autònoma de Barcelona, Bellaterra, Barcelona, Spain; 2 Department de Genètica i de Microbiologia, Universitat Autònoma de Barcelona, Bellaterra, Barcelona, Spain; 3 CIBER de Bioingeniería, Biomateriales y Nanomedicina (CIBER-BBN), Bellaterra, Barcelona, Spain; 4 CSIR-Institute of Genomics and Integrative Biology, University Campus, Mall Road, Delhi 110007, India; 5 Laboratory of Immunology, Regional Norte, Universidad de la Republica, Gral. Rivera 1350; Salto, 50.000, Uruguay; 6 CSIR-Indian Institute of Toxicology Research, Mahatma Gandhi Marg, Lucknow 226001, Uttar Pradesh, India

## Abstract

Histidine-rich peptides are commonly used in recombinant protein production as purification tags, allowing the one-step affinity separation of the His-tagged proteins from the extracellular media or cell extracts. Genetic engineering makes feasible the post-purification His-tag removal by inserting, between the tag and the main protein body, a target site for trans-acting proteases or a self-proteolytic peptide with regulatable activities. However, for technical ease, His tags are often not removed and the fusion proteins eventually used in this form. In this commentary, we revise the powerful biological properties of histidine-rich peptides as endosomolytic agents and as architectonic tags in nanoparticle formation, for which they are exploited in drug delivery and other nanomedical applications. These activities, generally unknown to biotechnologists, can unwillingly modulate the functionality and biotechnological performance of recombinant proteins in which they remain trivially attached.

## Affinity protein purification

Protein engineering and production, as a widely spread methodological platform, is providing reagents for catalysis, drugs for pharmaceutical industries and in general, instruments for analytical research in structural biology, proteomics, interactomics and drug design, among others. If secreted, recombinant proteins produced in prokaryotic or eukaryotic cell factories are found diluted in the medium, or alternatively, they occur as minor components of extremely complex macromolecular mixtures if retained inside the cell. Therefore, they must be purified so as achieve conveniently high concentration and purity levels required for stable storage and characterization or for efficient use in biological systems or in catalysis. The identification of peptide tags for single-step affinity-based purification, upon end terminal fusion, has provided an advantageous tool that represents a faster, easy and cost-effective alternative to multiple-step chromatographic separations [1,2]. Protein tagging allows the purification of virtually any protein without previous knowledge of its properties or those of its expected contaminants, since the capturing event relies on tag's rather than on protein's features.

Being absent in the natural target protein, affinity tags are expected to be removed upon purification, to keep the target protein sequence as natural as possible [3,4]. During the upstream cloning strategies, recombinant proteins can be designed to contain target sites for trans-acting proteases [4] or alternatively, functional endoproteolytic peptides [5] to remove the additional peptides. The Tobacco Etch Virus TEV protease (TEVp) is probably the most used agent for *trans *tag removal, because of its high specificity for the TEVp target site (ENLYFQ↓G) compared to the most promiscuous behaviour of alternative enzymes such as factor Xa, enterokinase and some picornaviridae 3C proteases [4]. On the other hand, inteins are among the most used *cis*-acting tag-removal agents [6-8].

However, for technical convenience, the foreign peptide is often not removed after purification, as for instance, the C-position of the cleavage site in the recognition sequence of most endoproteases makes specially difficult the complete removal of C-terminal added stretches [1]. Obviously, the modification of the primary amino acid sequence is expected to have effects on physicochemical parameters of the protein such as the distribution of charged amino acids and the final conformation, and also on biological properties such as in intra- or intermolecular interactions, solubility and enzymatic activity. However, some observations suggest that such effects might be extremely soft or even convenient. In this context, a few additional end terminal amino acids seem not affecting the packing of the crystals or atomic interactions, leading to the correct 3D structure determination [9]. Furthermore, the presence of purification tags might positively affect the solubility, productivity and proteolytic stability of recombinant proteins [10-14], which would provide an added value to these protein segments. These observations have prompted researchers to evaluate affinity tags not only for their usefulness as tools for downstream but also as midstream folding-assistant agents [1]. Finally, some analytical procedures to determine *in vivo *protein-protein interactions (such as the tandem affinity purification) are based on the presence in the model protein of affinity purification tags [15,16], relying on the assumption that these additional segments do not dramatically influence the protein's natural capability to perform cross-molecular interactions.

## Histidine-rich peptides in Biotechnology

Histidine-rich peptides (often H6 but also other peptide versions, Table 1) are probably the most used protein purification tags [1]. Being short sequences, His-tags do not add significant metabolic load to the protein production process and they can be easily incorporated to the protein by simple genetic engineering at the upstream level. Since the interaction between a His-tag and its ligand does not require any specific conformation, binding is feasible under either native or denaturing conditions (e.g. urea 8 M or guanidinium hydrochloride 6 M) [17] as well as under oxidizing or reducing conditions, resulting in high technological flexibility and easy scalability [18].

**Table 1:** Representative examples of His-rich peptides used in biotechnology as affinity tags for protein purification.

Protein	Tag sequence	Position in the fusion protein	Tag removal	Use or biological activity	# of His residues	Reference or PDB entry
GFP	H6-N1-H6	C	No	Protein immobilization	12	[35]

N-acetiltransferase (PA4794)	GSS-H6	N	No	Putative antibiotic resistance protein	6	3KKW

Toxoflavin-degrading enzyme (TxDE)	H8	C	No	Phytotoxin degradation	8	[36]

Affibody	HEHEHE	N	No	*In vivo *imaging	3	[37]

Antibody	H6	C	No	Antibody Against HER3 and EGFR	6	[38]

Low-density lipoprotein receptor-related protein 6	H8	N	No	Blood brain barrier crossing	8	[39]

eEF-2K	thioredoxin-H6	N	Yes	Modulation of rate of protein synthesis	6	[40]

Human peripheral cannabinoid receptor (CB2)	H10	C	Yes	Intracellular signal transduction	10	[41]

Serine protease granzyme B (grB)	H6	N	Yes	Apoptosis inducer	6	[42]

Human granulocyte macrophage colony stimulating factor (hGMCSF)	H6	N	Yes	Cytokine	6	[43]

Immobilized metal affinity chromatography (IMAC) [19] is a separation principle based on the differential and reversible affinity of transition metal ions such as Zn^2+^, Cu^2+^, Ni^2+^, and Co^2+ ^for histidines [20], due to the coordination bonds formed between metal ions and amino acid side chains exposed on the protein surface. Electron-donor atoms (N, S, O) present in the chelating compounds of the chromatographic support are capable of coordinating metal ions and forming metal chelates, which can be bidentate, tridentate, etc., depending on the number of occupied coordination bonds [21]. Four of the six coordination sites on the octahedral Ni^2+ ^center are occupied by the nitrilotriacetic acid (NTA) ligand, and the remaining coordination sites are occupied by two of the six imidazole moieties in the H6-tag. The model in which the interaction of the metal ion with His-tag locates in residues n and n+2 of the tag is confirmed by the fact that IMAC ligands can bind to His-tags consisting of consecutive as well as to alternating histidine residues.

As the chelating ligand is used to fix the metal to agarose, iminodiacetic acid (IDA) was initially employed and it is still in use today in many commercial IMAC resins. This technology was improved by the invention of the chelating ligand NTA in the 1980s [22].

The amino acid histidine shows the strongest interaction with immobilized metal ion matrices, as electron donor groups on histidine imidazole ring readily form coordination bonds with the immobilized metal. In Biacore experiments, the dissociation rate of a H6-tagged protein to Ni-NTA was estimated to be 1 × 10^-6 ^to 1.4 × 10^-8 ^M at pH 7.0-7.4. The same study also showed that two histidines separated by either one or four other residues, are the preferred binding motifs [23]. Interestingly, selection of an optimum tag by a phage-displayed library showed that tags with only two histidine residues possessed chromatographic characteristics superior to those of the most commonly used H6-tag [24]. Adsorption of a protein to the IMAC support is usually performed in neutral or slightly basic media in which imidazole nitrogens in histidine residues are non-protonated. Elution of the target protein is achieved by protonation, ligand exchange or extraction of the metal ion by a stronger chelator, like EDTA.

## Histidine-rich peptides in Nanomedicine

In a very different context, histidine-rich peptides are largely exploited in drug delivery as powerful endosomal escape agents, by their incorporation into drug-loaded nanoparticles or polyplexes to be internalized by target cells through endosome formation. During centripetal intracellular trafficking, most of the uptake routes converge into endocytic vesicles, where both vehicle and cargo may become enzymatically degraded when late endosomes are transformed into lysosomes [25]. Within endosomes, the imidazole group of histidine, with a pK around 6.0, gets protonated under the increasingly acid conditions. This step recruits Cl^- ^ions with a consequent osmotic swelling and endosomal cracking (the 'proton sponge' model [26]), which results in the cytoplasmic release of the nanoparticle and escape from lysosomal enzymes. Histidine-rich peptides have been incorporated into polymers, liposomes and proteins (including virus-like particles, VLPs) (Table 2). In non-viral gene therapy, such histidine-empowered vehicles show up to almost 10^4^-fold transgene expression improvement when compared with their corresponding histidine-lacking, parental versions [27]. Despite other membrane-active peptides, poly-histidines remain neutrally charged at neutral pH, avoiding non specific binding to serum proteins and consequent inactivation of the particle [28].

**Table 2:** Representative examples of His-rich peptides used in nanomedicine as endosomolytic agents.

Type of construct	Tag sequence	Experimental model	# of His residues	References
pDNA/siRNA +peptide	CHK6HC CH3K3H3C CH6K3H6C	In vitro, HepG2, COS 7, and CHO cells, 10X more expression than w/o histidine	2-12	[44]

pDNA + peptide +lactosylated polylysine	H5WYG (23 aas; GLFHAIAHFIHGGWHGLIHGWYG)	In vitro, HepG2, B16 and Rb-1cells, 93-2150X more expression than control, with serum.	5	[45]
		
MS2 VLPs-peptide		In Hep3B cells mediate endosomal escape that doesn't occur without the peptide.	5	[46]
		
pDNA+ PEG-H5WYG		CHO cells; increase expression 2-5 fold.	5	[47]

Lipopeptide + pDNA	Lau/PalCK3H2	In vitro, COS 7 cells, similar results to PEI, lipofectamine	2	[48]

pDNA + peptide	LAH4 (26 aas; KKALLALALHHLAHLALHLALALKKA)	In vitro, human hepatocarcinoma cells, 10× more expression than lipofectamine.	4	[49]

Chitosan-CH + pDNA	Chitosan-CH Chit. KH dendron	In vitro, HEK293 cells, increases expression up to 50-fold over chitosan alone.	1	[50]
		
Chit. 4 gen KH dendron + pDNA		Chitosan dendron improves escape over Chitosan-CH	1	[51]

pDNA + peptide	Tat-H10 C-H5-Tat-H5-C	In vitro, in U251, H4, T98G and C6 cell lines, up to 7000-fold improvement. In vivo, in rat intrastriatum injection.	10	[27]
		
FuGENE lipid+ peptide+ pDNA		In vitro in 5 different cell lines, significant improvement over pDNA+ peptide alone	10	[52]

CM-PLH+PbAE+pDNA	Polymer CM-PLH	In vitro, in HEK293 and B16-F10 cells, and in vivo, i.v. mice injection; higher transfection efficiency over PbAE alone	1	[53]

STR-CH2R4H2C +pDNA	STR-CH2R4H2C	In vitro, COS-7 cells, improves lipofectamine levels of expression.	2	[54]

(KHKHKHKHKK)6-FGF2 + pDNA	KHKHKHKHKK	In vitro, NIH 3T3, T-47D and COS-1 cells; expression is detected in 15-41% of cells, but not quantitated; no serum.	24	[55]

Abbreviations: aas, amino acids; pDNA, plasmid DNA; PEI, poly (ethylene imine); aas, aminoacids; Lau, lauryl; Pal, palmytoil; chit. 4 gen, chitosan 4 generation; CM-PLH, carboxymethyl poly (L- histidine); PbAE, poly (β-amino ester); STR, staeroyl; FGF-2, fibroblast growth factor-2.

The number of histidines and their distribution in the amino acid sequence of the tag determine endosomal escape efficiency of His-tagged vehicles and nanoparticles. Lo and Wang [27] showed that the endosomolytic activity of Tat-H10 fusion peptides could be 10-fold higher than Tat-H5 and Tat-H20 versions. Also, 10 His residues distributed at both sides of Tat peptide (C-5H-Tat-5H-C), are 10^2^-fold more efficient than its equivalent C-Tat-10H-C. Although the optimal number of His residues is believed to be ranging between 5 and 10 (similar to the figures seen in His-based affinity tags, Table 1), the incorporation of only one His residue is sufficient to grant membrane-active properties to a drug delivery system [29]. Interestingly, although most of His-tags seem to favour intracellular targeting exclusively through proton sponge activities, LAH4 (KKALLALALHHLAHLALHLALALKKA) is believed to interact with the endosomal membrane at pH 7, due to its amphipathic nature, and disrupt its physical integrity upon subsequent protonation [30].

On the other hand, H6 has been recently found involved in intermolecular protein-protein interactions, leading to the self-assembling of C-terminal His-tagged building blocks into nanoparticles, useful as vehicles in non viral gene therapy [31,32]. The nature of such organizing interactions is yet to be completely understood, and might involve other specific amino acids such arginines. Since the resulting nanoparticles seem to be more efficient in transgene expression when formed at slightly acidic pH values [32], its seems reasonable to speculate that protonated, overhanging H6 tails interact with anionic protein areas of siding building bocks and also with DNA. A deeper understanding of the interactions promoted by His-rich peptides could offer tools for the rational engineering of nanoparticle formation and DNA encapsulation, both issues of critical relevance in bionanomedicine [33,34].

## Conclusions

Protein production scientists are usually unaware of the potent biological properties of histidine-rich peptides for which they are used in nanomedicine (essentially endosomal disruption but also cross-molecular electrostatic interactions), which are activated at acidic pH. The same (or very similar) His-rich peptides used in affinity chromatography, incorporated into different type of nanoparticles (most ranging from H2 to H10, Table 2) to favour intracellular trafficking in drug delivery [25], are equally employed as affinity tags for separation and often not removed after protein purification (Table 1). These remaining agents might render unexpected protein behaviour, when exposed to cells or nucleic acids, and specific measures for control should be implemented in biological assays when technical obstacles make His-tag removal inconvenient upon protein purification.

## Competing interests

The authors declare that they have no competing interests.

## Authors' contributions

NFM and JLC have equally contributed to this manuscript. All authors have read and approved the final version.
